# Antifungal Potential of *Melaleuca alternifolia* against Fungal Pathogen *Fusarium oxysporum* f. sp. *cubense* Tropical Race 4

**DOI:** 10.3390/molecules28114456

**Published:** 2023-05-31

**Authors:** Pavitra Paramalingam, Nadiya Akmal Baharum, Janna Ong Abdullah, Jeum Kyu Hong, Noor Baity Saidi

**Affiliations:** 1 Department of Cell and Molecular Biology, Faculty of Biotechnology and Biomolecular Sciences, Universiti Putra Malaysia, Serdang 43400, Selangor, Malaysia; 2 Division of Horticultural Science, Gyeongsang National University, 33 Dongjin-ro, Jinju 52725, Republic of Korea; 3 Agri-Food Bio Convergence Institute, Gyeongsang National University, 33 Dongjin-ro, Jinju 52725, Republic of Korea; 4 Laboratory of Sustainable Agronomy and Crop Protection, Institute of Plantation Studies, Universiti Putra Malaysia, Serdang 43400, Selangor, Malaysia

**Keywords:** tea tree, essential oil, hydrosol, TR4, banana, bioprotectant

## Abstract

Fusarium wilt of bananas caused by *Fusarium oxysporum* f. sp. *cubense* Tropical Race 4 (Foc TR4) poses the most serious threat to banana production globally. The disease has been managed using chemical fungicides, yet the control levels are still unsatisfactory. This study investigated the antifungal activities of tea tree (*Melaleuca alternifolia*) essential oil (TTO) and hydrosol (TTH) against *Foc* TR4 and their bioactive components. The potential of TTO and TTH in inhibiting the growth of *Foc* TR4 was evaluated in vitro using agar well diffusion and spore germination assays. Compared to the chemical fungicide, TTO effectively suppressed the mycelial growth of *Foc* TR4 at 69%. Both the minimum inhibitory concentration (MIC) and minimum fungicidal concentration (MFC) of TTO and TTH were established at 0.2 µg/µL and 50% *v*/*v*, respectively, suggesting the fungicidal nature of the plant extracts. The disease control efficacies were also demonstrated by a (*p* ≤ 0.05) delayed Fusarium wilt symptom development in the susceptible banana plants with reduced LSI dan RDI scores from 70% to around 20–30%. A GC/MS analysis of TTO identified terpinen-4-ol, eucalyptol, and α-terpineol as the major components. In contrast, an LC/MS analysis of TTH identified different compounds, including dihydro-jasmonic acid and methyl ester. Our findings indicate the potential of tea tree extracts as natural alternatives to chemical fungicides to control *Foc* TR4.

## 1. Introduction

The soil-borne fungus *Fusarium oxysporum* f. sp. *cubense* (*Foc*) is the cause of major losses to banana plantations globally [1,2]. Based on a recent projection, 17% of today’s banana-growing area could be lost over the next 20 years due to the rapid spreading of Fusarium wilt disease [3]. *Foc* can survive in the soil for a very long time by producing chlamydospores that can withstand desiccation and an unfavorable environment, thus presenting a great challenge in controlling the disease once it is established [4]. Among the *Foc* races, Tropical Race 4 (TR4) of *Foc* is considered the most virulent [5].

Disease control approaches, including physical, chemical, and biological approaches, have been practised singly or in combination with other disease management strategies to prevent and manage Fusarium wilt in bananas [6]. Among them, the application of chemical fungicide is by far the most adopted strategy despite being marginally effective [7,8]. However, the continuous use of chemical fungicides can lead to fungal resistance and is harmful to human health in the long term.

On the other hand, biological control offers a promising disease management approach due to its safety and smaller environmental impact [9,10]. Biological control methods include the use of living organisms, non-living extracts, and natural products. Natural plant extracts have shown great potential in limiting pathogen development, inducing plant defence responses, and modulating plant growth [11]. They are effective as bioprotectants against a broad spectrum of plant pathogenic fungi such as *Fusarium solani, F. graminearum, F. oxysporum, Alternaria solani, A. alternata*, and *Botrytis cinerea* [12,13,14,15]. The plant extracts can either be fungicidal (killing pathogens) or fungistatic (limiting pathogen development) in nature [16].

Tea tree (*Melaleuca alternifolia)* is a non-native Malaysian medicinal plant known for its antimicrobial, antibacterial, and antioxidant properties [17]. Recently, the plant has attracted increasing attention for its bioprotectant function against fungal pathogens, including *Foc* race 1 [18,19,20]. Foliar spray with TTO on infected banana plants in the field systematically induced protection against *Foc* race 1 in the daughter plants [19]. Interestingly, the observed effects surpassed the treatment by a triazole-based fungicide used as a positive control in the study. In another study, TTO was applied as a complex with polymer β-cyclodextrin, which improved its thermal stability and inhibited its volatility and oxidation [21]. The β-cyclodextrin–tea-tree complex successfully inhibited the growth of *B. cinerea* in vitro and reduced the incidence of fruit rot on tomatoes. TTO usually occurs as a combination of various types of bioactive compounds with different polarities, including monoterpene hydrocarbons, monoterpene hydrocarbon oxygenated derivatives, and a small number of sesquiterpenes [22]. 

Compared to essential oil, hydrosol, which is considered a by-product of the hydrodistillation process, contains a lower concentration of active compounds. However, the potential of hydrosols as antimicrobial and antifungal agents has also been reported previously [23,24]. For example, Belabbes et al. [24] showed that both the essential oil and the hydrosol of *Calendula arvensis* L. have antifungal activity against *Penicillium expansum* and *Aspergillus niger*, but the latter exhibited better antifungal activity. Previously, we have also shown that tea tree hydrosol (TTH) effectively suppressed the mycelial growth of *Foc* TR4 in vitro [25]. We hypothesized that TTO would have a stronger antifungal effect against *Foc* TR4 due to its higher concentration of active compounds. Therefore, this work aimed to investigate the bioprotectant effects of TTO against the banana pathogen *Foc* TR4 in vitro via agar well diffusion and spore germination assay. In addition, the in vivo antifungal activity of TTO and TTH were tested by applying the extracts as a treatment to two susceptible banana cultivars, Berangan and Cavendish prior to inoculation with *Foc* TR4. At the end of the study, both extracts were analyzed for their bioactive components.

## 2. Results

### 2.1. In Vitro Antifungal Activity of Tea Tree Essential Oil and Hydrosol against Foc TR4 

**Figure 1:**
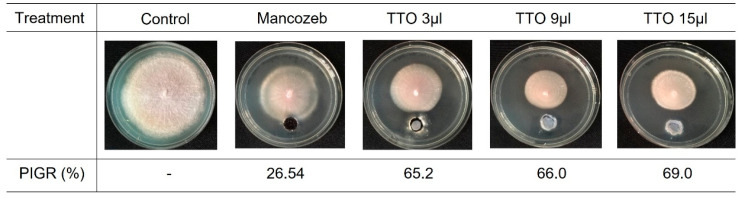
Inhibition of mycelial growth of *Foc* TR4 by different volumes of tea tree essential oil after eight days of incubation.

**Figure 2:**
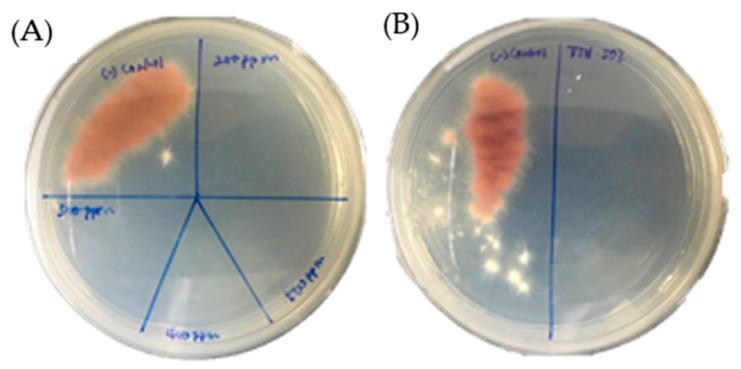
Determination of minimum fungicidal concentration (MFC) of TTO (**A**) at 0.2, 0.3, 0.4, and 0.5 µg/µL and TTH (**B**) at 50% (*v*/*v*) on *Foc* TR4 growth after five days of incubation.

The agar well diffusion assay revealed that the mycelial growth of Foc TR4 was suppressed in all treated plates, with the maximum PIGR at 69% observed in plates treated with 15µL TTO, compared to the untreated. (Figure 1). The chemical fungicide mancozeb at 5 mg/mL was less effective than TTO in suppressing the mycelial growth of Foc TR4. The MIC of TTO that resulted in complete growth inhibition of Foc TR4 was 0.2 µg/µL (Figure S1 in Supplementary Materials). Whereas for the TTH, the MIC was observed at 50% (*v*/*v*). The MFC for TTO and TTH were also recorded at 0.2 µg/µL and 50% (*v*/*v*), respectively (Figure 2). 

**Figure 3:**
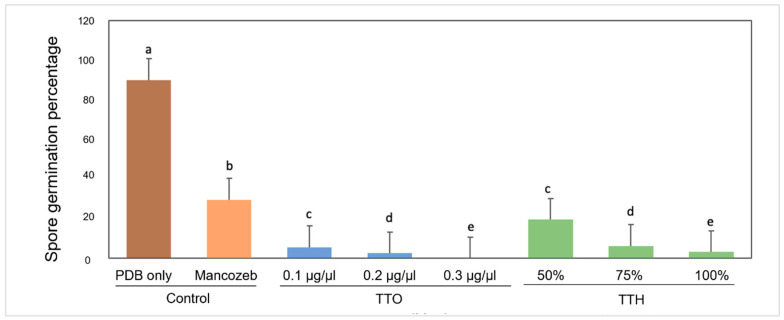
Antifungal efficacies of TTO and TTH against spore germination of *Foc* TR4. Values shown are means ± SE (*n* = 3). Columns denoted by different letters indicate significant difference according to Tukey’s test.

The fungal spore germination was significantly (*p* ≤ 0.05) inhibited by TTO and TTH at all concentrations 24 h after the treatment (Figure 3). The effect of mancozeb on *Foc* TR4 spore germination was weaker than the tea tree extract’s. At a concentration of 0.3 µg/µL, TTO completely inhibited fungal spore germination, while the undiluted TTH (100%) only reduced the spore germination to 3.3%. 

### 2.2. In Vivo Antifungal Activity of Tea Tree Essential Oil and Hydrosol against Foc TR4

**Figure 4:**
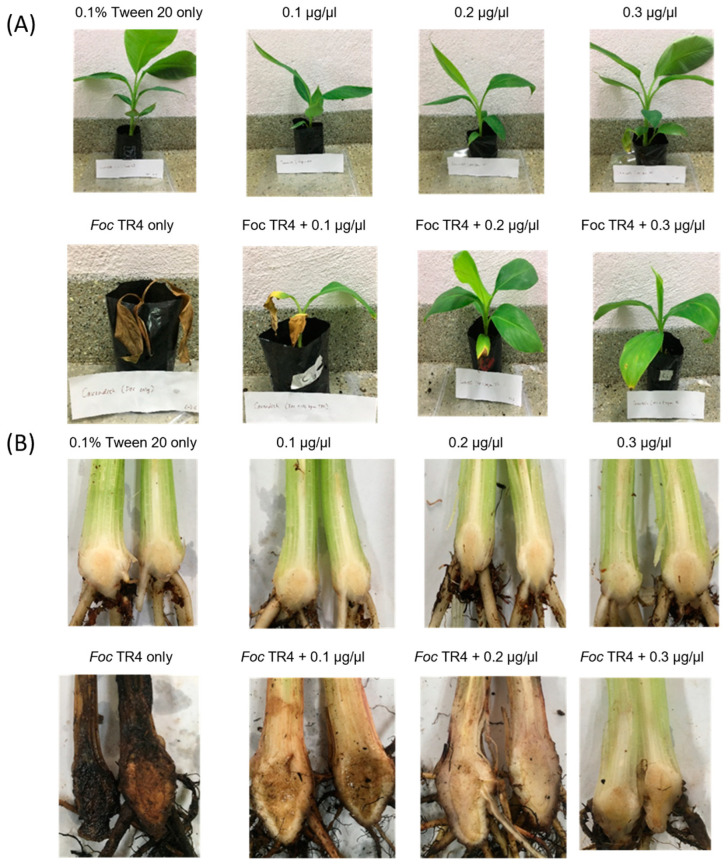
Phenotypes of LSI (**A**) and RDI (**B**) on Cavendish plantlets following inoculation with *Foc* TR4 at four weeks post-treatment with TTO (0.1, 0.2, and 0.3 µg/µL). Control plants received 0.1% Tween^®^ 20.

Cavendish plantlets treated with TTO showed moderate external symptoms, indicated by yellowing and necrosis of the lower leaves at 0.1 and 0.2 µg/µL. In comparison, only slight yellowing of lower leaves was observed at 0.3 µg/µL (Figure 4A). On the other hand, plantlets infected with *Foc* TR4 without any treatment demonstrated severe external symptoms where the leaves wilted entirely and eventually abscised, leading to the plant’s death. 

To determine the severity of Fusarium wilt disease based on internal discoloration, the rhizomes were dissected (Figure 4B). Cavendish plantlets inoculated with *Foc* TR4 without treatment exhibited dark discoloration of the entire rhizome stele. In contrast, TTO treatment reduced internal symptoms, as evidenced by the lesser discoloration of tissues in the stellar region of the treated plantlets. The uninoculated plantlets appeared healthy throughout the bioassay. The antifungal effects of TTH on Cavendish plantlets in vivo were similar to those of TTO (Figure S2), and the effects were replicated in the Berangan cultivar (Figures S3 and S4). 

**Figure 5:**
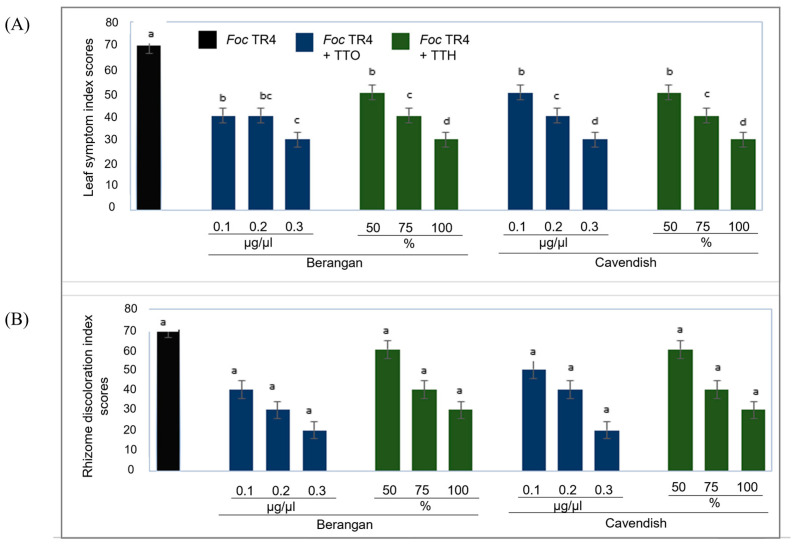
Effects of TTO and TTH on the severity of Fusarium wilt in Berangan and Cavendish plantlets based on (**A**) leaf symptom index (LSI) and (**B**) rhizome discoloration index (RDI). Values shown are means ± SE (*n* = 5). Statistical data analysis was carried out by SPSS software using Tukey’s test. Values with different letters are significantly different (*p* ≤ 0.05).

Irrespective of cultivars, banana plantlets infected with *Foc* TR4 without treatment recorded LSI and RDI scores of 70% (Figure 5). Conversely, the LSI score for treated plantlets was reduced to 30%, whereas for RDI, the score was as low as 20%. However, the RDI was not statistically significant compared to LSI in this study. Notably, no phytotoxic effects were observed on the banana plantlets that only received tea tree extracts. 

### 2.3. Yield and Composition of Tea Tree Essential Oil

In this study, the yield of TTO was recorded at 2.2%. The GC/MS analysis of TTO detected fourteen compounds with over 80% similarity with the reference standard found in the NIST library (Table 1, Figure S5). The identified compounds mostly belong to the terpene chemical family. The TTO was characterized by monoterpenoids, with terpinen-4-ol (22.98%) having the highest percentage area, followed by eucalyptol (20.46%). The monoterpene groups mainly consisted of ç-terpinene (7.31%) and benzene-2-ethyl-1,3-dimethyl (7.31%). 

### 2.4. Yield and Composition of Tea Tree Hydrosol

**Figure 6:**
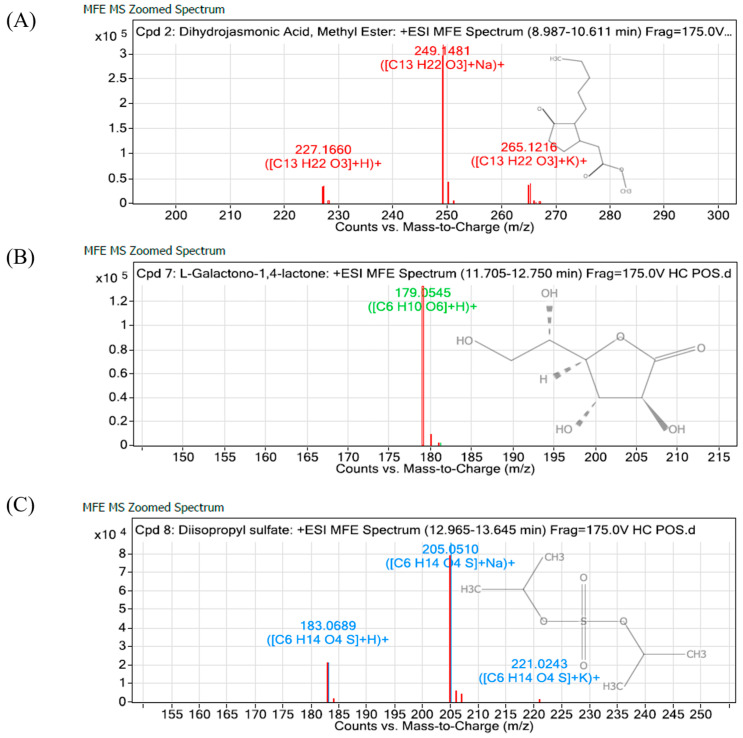
Chemical profiles (total ion current) of TTH obtained by QTOF-LC/MS analysis.

The yield recorded for TTH was slightly higher than TTO at 25%, but only three compounds were detected from the analysis using the positive ionization mode (Table 2). Among the known compounds, the dihydro-jasmonic acid methyl ester (3) was the most abundant. The chromatograms of all the detected compounds are shown in Figure 6. 

## 3. Discussion

The biological control approach using microbes or natural plant extracts in managing the Fusarium wilt of bananas is the viable option compared to chemical control using fungicides or fumigants [7]. Tea tree extracts have been proven to have broad-spectrum antifungal activity against a wide range of plant fungal pathogens [26,27,28] and to be effective even at a low concentration. In the study by Terzi et al. [26], a single foliar treatment containing TTO as low as 0.5% was effective in preventing *Blumeria graminis* from colonizing barley leaves. Apart from acting directly on the pathogen, the antifungal effects of TTO can indirectly induce defense mechanisms in treated plants, as evident from the induction of pathogenesis-related genes in TTO-treated banana plants [19]. Previously, we have established the antifungal activity of the less-studied TTH on *Foc* TR4 in vitro [25]. In the present study, we showed that TTO exhibited strong antifungal activity against *Foc* TR4 in vitro by suppressing mycelial growth by more than 50%. Interestingly, the suppressive effects surpassed the chemical fungicide mancozeb. The fungicidal activity of TTO can be related to its capacity to disrupt cell membranes and cell walls of fungi [20,29].

In general, plant extracts can either be fungicidal (killing pathogens) or fungistatic (limiting pathogen development) in nature [16]. The recorded MIC and MFC values for TTO (0.2 µg/µL) and TTH (50%) were identical, suggesting that the extracts are fungicidal [30]. The low MIC values also indicate that the extracts exhibit high antifungal potency against *Foc* TR4. At 0.3 µg/µL, TTO completely inhibited *Foc* TR4 spore germination, while undiluted hydrosol only gave a minor effect. This may be due to the differences in the chemical constituents of the two extracts. Zatla et al. [23] reported similar findings where the essential oil of *Daucus carota* effectively inhibited the sporulation of *Penicillium expansum and B. cinerea* compared to the plant hydrosol. According to their study, *D. carota* essential oil contains higher amounts of constituents such as oxygenated diterpenes and sesquiterpenes than its hydrosol.

The effects of TTO and TTH on the development of Fusarium wilt disease were further analyzed in Berangan and Cavendish cultivars that are highly susceptible to *Foc* TR4 infection in the tropics and subtropics [31]. The extracts similarly reduced the LSI dan RDI scores from 70% to 20–30% in both cultivars. Previously, the crude extracts of *Allium tuberosum* reduced the disease severity index by 81% in the Cavendish clone ‘Baxi’ infected with *Foc* TR4 [32]. Collectively, these results indicate that natural plant extracts have a great potential to control *Foc* TR4.

In terms of the bioactive compounds present in TTO, a similar profile was obtained as reported in other studies but with different percentages. The difference could be attributed to geographical location and climatic conditions in tropical countries. Most of the main components of TTO are well-studied, especially the terpinen-4-ol, which was reported to show strong antifungal activity [12,33,34]. On the other hand, this study is the first to characterize components of TTH via LC/MS. Only three compounds were successfully identified in this study. Dihydro-jasmonic acid (a plant hormone belonging to the jasmonic acids, JA group) and methyl esters, which are the most abundant known compounds in TTH, have been shown to be involved in plant defence against various microbial pathogens. For example, the extracts of *Rhanterium epapposum* dominated by methyl esters have been shown to be effective against different types of bacterial and fungal pathogens [35]. On the other hand, accumulation of JA in plants was observed in broad bean plants that exhibited increased resistance to infection by the bean rust pathogen [36]. More recently, an increase in JA production was reported to contribute to the *Arabidopsis thaliana* resistance toward *B. cinerea* [37]. The application of dihydro-jasmonic acid as an agent for disease tolerance in plants has been patented [38], suggesting the significant role of the compound in promoting plant immunity against pathogen infection.

## 4. Materials and Methods

### 4.1. Plant and Fungal Materials

The fresh leaves of the tea tree were harvested from the Conservatory Park, Universiti Putra Malaysia, from October to December 2019 separately at the latitude of 3°00′01.0″ N and longitude of 101°43’19.3” E. The taxonomical identification of the species was verified by Dr. Shamsul Khamis of the Biodiversity Unit, Institute of Bioscience, Universiti Putra Malaysia, Serdang, Selangor, Malaysia, and deposited in the Herbarium of the Biodiversity Unit with voucher number SK 2224/13. Banana cultivars used in this study were *Musa accuminata* AAA cultivar Cavendish and Berangan. Hardened two-month-old Berangan and Cavendish banana plantlets were purchased from Felda Sungai Tekam, Jerantut, Pahang. *Fusarium oxysporum* f. sp. *cubense* Tropical Race 4 strain C1HIR_9889 (GCA_001696625.1) isolated from Terengganu, Malaysia (VCG type 01213/16) was obtained from the University of Malaya, Kuala Lumpur.

### 4.2. Isolation of Essential Oil and Hydrosol 

Isolation of TTO and TTH was done according to [39] at a 2:1 solvent-to-solid ratio. The essential oil (top layer) and hydrosol (lower aqueous layer) were separated using a separatory funnel and stored at 4 °C in closed vials. A fresh weight basis (*w*/*w*) was used to estimate the yield of plant extracts (in percentage). The essential oil and hydrosol were directly used in the experiment without dilution unless mentioned. 

### 4.3. Agar Well Diffusion Assay

Different amounts of TTO (3, 9, and 15 µL) were added separately into the 9.5 mm diameter wells on potato dextrose agar (PDA) (Oxoid, United Kingdom) (25 mL) in individual petri dishes [25]. PDA without TTO served as the negative control, while PDA with 5 mg/mL of chemical fungicide mancozeb (Behn Mayer AgriCare (M) Sdn Bhd, Malaysia) was used as the positive control. Five-day-old *Foc* TR4 mycelial discs (5 mm) were cut from the edge of a growing fungal colony and used to inoculate all plates. The plates were incubated in the dark at RT. The diameter of *Foc* TR4 was measured daily until the fastest-growing mycelium reached the edge of the plate. The percentage inhibitory of radial growth (PIRG) was calculated using the following formula: PIRG% = [(R1 − R2)/R1] × 100%, where R1 = radial growth in the negative control plate and R2 = radial growth in the treatment plate.

### 4.4. Determination of Minimum Inhibitory Concentration (MIC) and Minimum Fungicidal Concentrations (MFC) 

The MIC of TTO and TTH was determined by broth macrodilution assay based on [40]. Different concentrations of TTO ranging from 0.1 to 0.5 µg/µL emulsified with 0.01% (*v*/*v*) Tween^®^ 20 and TTH ranging from 10 to 50% (*v*/*v*) were prepared in 10 mL potato dextrose broth (PDB) (Oxoid, United Kingdom) in 15 mL plastic conical tubes. The spore or conidia suspension of *Foc* TR4 was prepared by adding 10 mL of sterile water to the mycelium growing on PDA plates for five days. The flooded mycelium was then scraped using a sterile glass rod, and the suspension was passed through a funnel plugged with cotton wool. The collected conidia were adjusted to 10^6^ conidia/mL using a hemacytometer. The same method of spore suspension preparation was followed for the rest of the experiments. The PDB was inoculated with 10 µL of *Foc* TR4 spore suspension and incubated for six days at 28 °C. PDB without TTO and TTH served as the negative control, while PDB with 5 mg/mL of mancozeb served as the positive control. The lowest concentration of TTO and TTH at which there was 100% inhibition (no growth) was considered the MIC. To establish the MFC, 10 µL of PDB were removed from the inoculated conical tubes without any visible growth, spotted onto PDA, and kept at 28 °C. The MFC is the lowest tea tree extract concentration that kills the fungus. Each experiment was carried out in three replicates. 

### 4.5. Spore Germination Assay

TTO at different concentrations (0.1, 0.2, and 0.3 µg/µL) were emulsified with 0.1% (*v*/*v*) Tween^®^ 20 (Sigma-Aldrich, Burlington, MA, USA) and TTH (50, 75, and 100 % (*v*/*v*)) were prepared in PDB. Then, 100 µL of the TTO and TTH from each concentration were aliquoted onto a concave glass slide, followed by 30 µL of *Foc* TR4 spore suspension at 10^6^ conidia/mL. The slides were incubated at 28 °C for 24 h. Then, the slides were stained with lactophenol cotton blue stain (Hardy Diagnostics, Santa Maria, CA, USA) and observed under 100× magnification with a light microscope (Olympus, Shinjuku, Tokyo, Japan). Experiments were carried out in three replicates. The spore germination percentage was calculated as % SNG= SNG/SG + SNG × 100 [%SNG: percentage of non-germinated spores, SG: number of germinated spores, and SNG: number of non-germinated spores]. 

### 4.6. In Vivo Antifungal Assay 

Banana plantlets were removed from polybags and inoculated with *Foc* TR4, according to [41]. Briefly, the uprooted plantlets were washed and wounded by gently crushing the root tip using a forcep before inoculation. The plantlets were then dipped into a beaker containing the *Foc* TR4 conidia suspension at 10^6^ conidia/mL and incubated for two hours. Meanwhile, polybags containing 800 g commercial garden soil were treated with 50 mL of TTO in 0.1% Tween^®^ 20 (0.1, 0.2, and 0.3 µg/µL) or TTH (50%, 75%, and 100% (*v*/*v*)) via soil drenching [42]. Then, the inoculated plantlets were re-planted into the polybags containing the treated soils. Banana plantlets that received TTO or TTH treatment without *Foc* TR4 or were only treated with *Foc* TR4 served as control. A total of three biological replicates were used in each treatment. Disease severity was observed and recorded every week for one month. The leaf symptom disease (LSI) and rhizome discoloration index (RDI) were scored using the following formula, DI = [∑ (N_1-5_ × S_1-5_)/(N × S)] × 100%, where N_1-5_: number of symptomatic banana plants, S_1-5_: the score of symptoms, N: total number of banana plants, and S: the highest value of score of symptoms [43].

### 4.7. Gas Chromatography–Mass Spectrometry Analysis (GC/MS) of Tea Tree Essential Oil

The volatile constituent of TTO was analyzed using the Agilent 7890A GC system with Agilent 5975 mass selective detector (Agilent Technologies, Santa Clara, CA, USA). The MS was operated at electron impact ionization mode (ionization energy of 70 eV) and a mass range of m/z 20–1000. The GC system has an HP-5MS capillary column (30 m × 0.25 mm, film thickness 0.25 µm). The column temperature was initially held at 80 °C for 2 min, then increased to 240 °C at 5 °C/min, held for 5 min, and raised to 300 °Cat a rate of 3 °C/min and held for 5 min. The injector and detector temperatures were 225 °C and 300 °C, respectively. Gas chromatography was performed in the split mode with a split ratio of 20:1 with an injection volume of 1 µL for each diluted 0.01 g/mL extract. The carrier gas was helium and used at a 1.5 mL/min flow rate. Volatile components were identified by comparing the mass spectra of individual components and the reference mass spectra in the National Institute of Standards and Technology Mass Spectral Database library (NIST-MS 11).

### 4.8. Liquid Chromatography–Mass Spectrometry Analysis (LC/MS) Tea Tree Hydrosol

TTH was analyzed using the LC system composed of a quadrupole time-of-flight MS equipped with an electrospray ionization (ESI) source (Agilent Technologies, Santa Clara, CA, USA) that was used with an injection volume of 5 µL. Elution was performed on a column (100 × 2.1 mm, 2.6 µm) using solvents A (0.1% formic acid in water) and B (formic acid 0.1% in acetonitrile). The stepwise gradient from 5% to 100% solvent B was applied at a flow rate of 0.5 mL/min for 25 min. The autosampler and column oven temperatures were at 0.8 °C and 25 °C, respectively. A positive and negative ion mode was used with the mass range setting at m/z 100–1700. The ionization parameters were as follows: capillary temperature, 200 °C; spray voltage, 1000 V; and nebulizer sheath gas pressure, 35 PSI. 

### 4.9. Statistical Analysis

Data from in vitro and in vivo studies was analyzed and compared using a one-way ANOVA test at *p* ≤ 0.05 and regression analysis in SPSS software (Version 16.0) (Delaware, Chicago, IL, USA).

## 5. Conclusions

The antifungal effects of TTO and TTH were tested against the banana pathogen *Foc* TR4. TTO effectively suppressed the mycelial growth in vitro with a maximum PIGR of 69% and reduced the LSI and RDI in the susceptible banana plantlets to 30% and 20%, respectively. The in vivo antifungal activity of TTH was similar to that of TTO. Terpinen-4-ol was identified as the main compound in TTO, while dihydro-jasmonic acid methyl ester was among the most abundant compounds detected in TTH. Evidence shown in this manuscript supports the role of TTO and TTH as potent bioprotectant against *Foc* TR4 in bananas and can be considered an excellent control method to achieve sustainable agriculture goals. However, the application of essential oil as biocontrol is limited by its high volatility, low stability, and low water solubility. In the future, studies should be conducted to optimize TTO and TTH formulation for field applications to ensure maximum efficiency of disease control. In addition, the authorities should also consider improving the current policies to increase the adoption of this environmentally sensitive technology.

## Figures and Tables

**Table 1:** Chemical composition of tea tree essential oil (TTO).

Peak	Compound	Rt (s)	% Area	Chemical Family	Chemical Formula
**2**	á-Pinene	180.8	1.80	Monoterpene	C_10_H_16_
**3**	á-Myrcene	187.2	0.15	Monoterpene	C_10_H_16_
**4**	à-Phellandrene	200.4	0.18	Monoterpene	C_10_H_16_
**5**	p-Menth-4(8)-ene	210.5	2.89	Monoterpene	C_10_H_16_
**6**	Benzene, 1-methyl-3-(1-methylethyl)-	217.1	1.79	Monoterpenoid	C_10_H_14_S
**7**	Limonene	221.2	2.38	Monoterpene	C_10_H_16_
**9**	Eucalyptol	224.3	20.46	Monoterpenoid	C_10_H_18_O
**10**	Benzene, 2-ethyl-1,3-dimethyl-	249.3	7.32	Monoterpene	C_10_H_14_
**11**	γ-Terpinene	249.5	7.32	Monoterpene	C_10_H_16_
**14**	p-Menth-3-ene	280.5	0.38	Monoterpene	C_10_H_18_
**17**	Terpinen-4-ol	390.0	22.98	Monoterpenoid	C_10_H_18_O
**19**	à-Terpineol	405.5	4.86	Monoterpenoid	C_10_H_18_O
**22**	1,11-Hexadecadiyne	756.4	0.00		C_16_H_26_
**25**	Androstan-17-one, 3-ethyl-3-hydroxy-, (5à)-	836.2	0.56	Steroid	C_21_H_34_O_2_
**27**	Naphthalene, 1,2,3,5,6,8a-hexahydro-4,7-dimethyl-1-(1-methylethyl)-, (1S-cis)-	876.2	0.38	Sesquiterpene	C_15_H_24_

**Table 2:** Chemical composition of tea tree essential hydrosol (TTH).

Compound	RT (min)	*m/z* M-H^−^	Mass	Abundancy	Molecular Formula
Dihydro-jasmonic Acid, Methyl Ester	9.831	249.1481	226.1583	319,221.13	C_13_H_22_O_3_
L-Galactono-1,4- lactone	12.05	179.0545	178.047	133,254.17	C_6_H_10_O_6_
Diisoprop-yl sulfate	13.157	205.051	182.0618	86,138.21	C_6_H_14_ O_4_S

## Data Availability

The data presented in this study are available on request from the corresponding author.

## Supplementary Materials

The following supporting information can be downloaded at: https://www.mdpi.com/article/10.3390/molecules28114456/s1, Figure S1: Determination of minimum inhibitory concentration (MIC) of *TTO* (upper panel) and TTH (lower panel) against *Foc* TR4. Figure S2: Phenotype of LSI (A) and RDI (B) on Cavendish plantlets following inoculation with *Foc* TR4 with or without TTH treatment at four weeks post-treatment. Control plants received sterile distilled water. Figure S3: Phenotype of LSI (A) and RDI (B) on Berangan plantlets following inoculation with *Foc* TR4 with or without TTO treatment at four weeks post-treatment. Control plants received sterile distilled water. Figure S4: Phenotype of LSI (A) and RDI (B) on Berangan plantlets following inoculation with *Foc* TR4 with or without TTH treatment at four weeks post-treatment. Control plants received sterile distilled water. Figure S5: GC-MS peaks of *M. alternifolia* essential oil (TTO). Each peak represents the specific compound detected by analysis. X-axis represents the retention time (s) and Y-axis represents the concentration counts.

## Sample Availability

Samples of the compounds are not available from the authors.
