# Supplementary material for: Antifungal Potential of Melaleuca alternifolia against Fungal Pathogen Fusarium oxysporum f. sp. cubense Tropical Race 4
Source: Molecules. 2023 May 31;28(11):4456. doi: 10.3390/molecules28114456 (PMC10254191; doi:10.3390/molecules28114456)
Supplement: Supplementary file 1 [file molecules-28-04456-s001.zip › Figure S1.pdf]

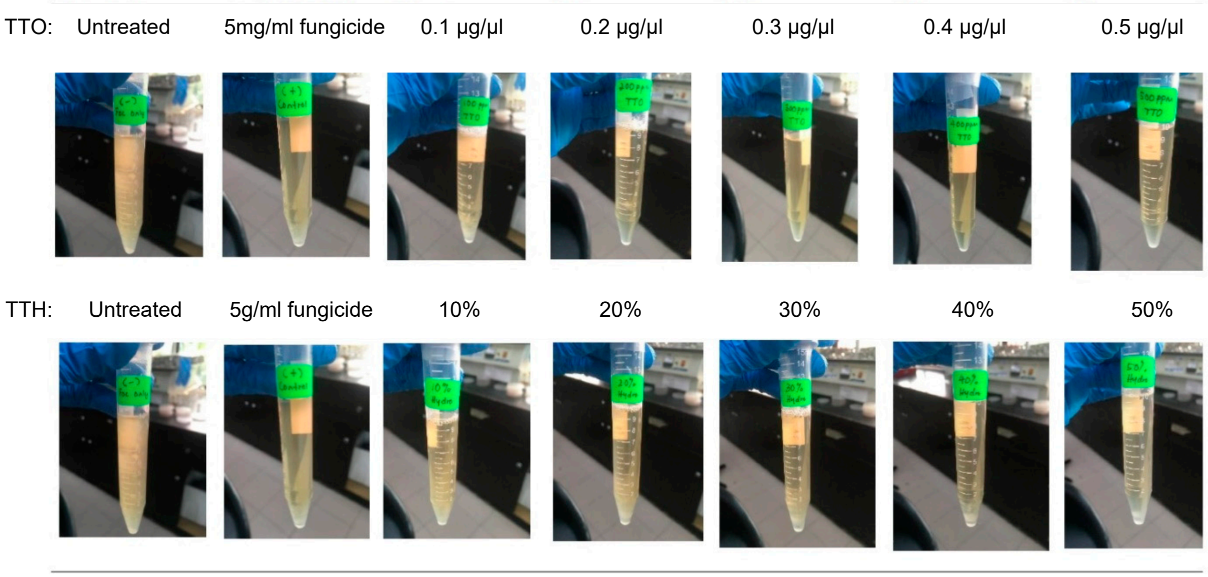

Figure S1 Determination of minimum inhibitory concentration (MIC) of *TTO* (upper panel) and TTH (lower panel) against *Foc* TR4.
