# Supplementary material for: Antifungal Potential of Melaleuca alternifolia against Fungal Pathogen Fusarium oxysporum f. sp. cubense Tropical Race 4
Source: Molecules. 2023 May 31;28(11):4456. doi: 10.3390/molecules28114456 (PMC10254191; doi:10.3390/molecules28114456)
Supplement: Supplementary file 1 [file molecules-28-04456-s001.zip › Figure S4.pdf]

A)

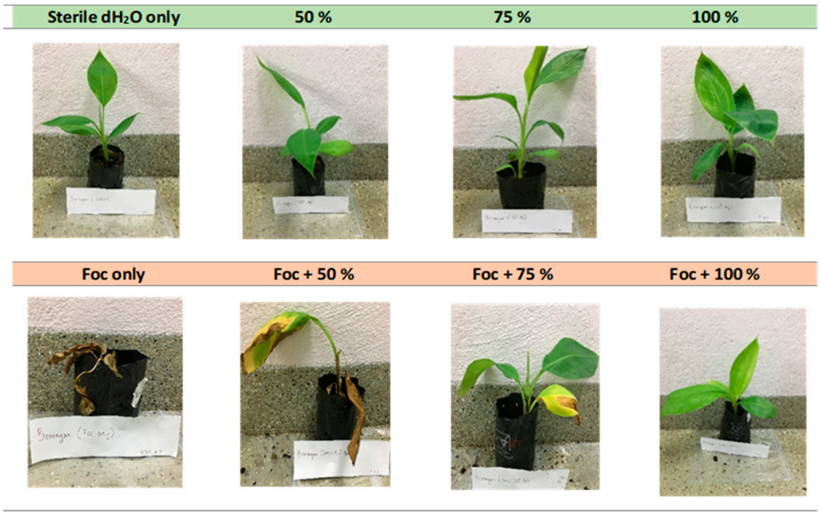

B)

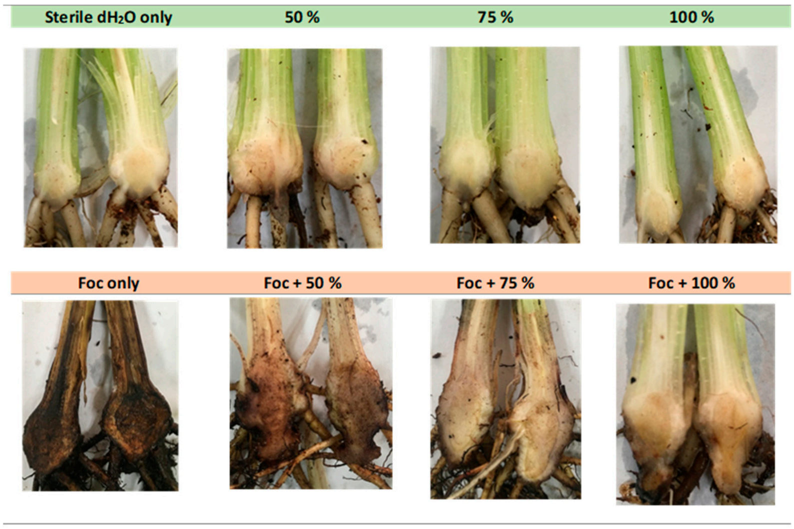

Figure S4: Phenotype of LSI A) and RDI B) on Berangan plantlets following inoculation with *Foc* TR4 with or without TTH treatment at four weeks post-treatment. Control plants received sterile distilled water.
