# Supplementary material for: Antifungal Potential of Melaleuca alternifolia against Fungal Pathogen Fusarium oxysporum f. sp. cubense Tropical Race 4
Source: Molecules. 2023 May 31;28(11):4456. doi: 10.3390/molecules28114456 (PMC10254191; doi:10.3390/molecules28114456)
Supplement: Supplementary file 1 [file molecules-28-04456-s001.zip › Figure S5.pdf]

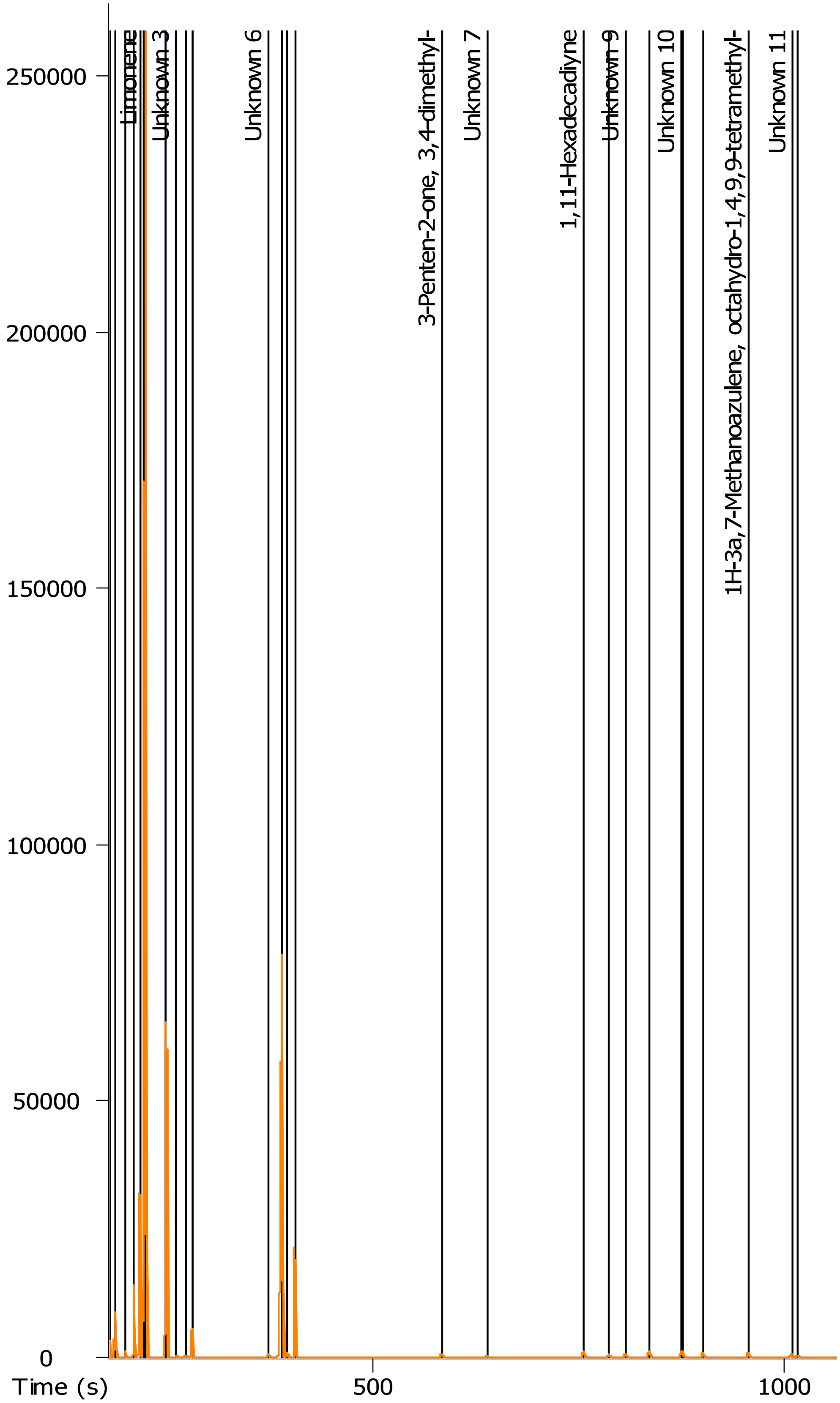

Figure S5 GC-MS peaks of M. alternifolia essential oil (TTO). Each peak is representing the specific compound detected by analysis. X-axis represents the retention time (s)and Y-axis represents the concentration counts.
